# LC-ESI-LTQ-Orbitrap-MS for Profiling the Distribution of Oleacein and Its Metabolites in Rat Tissues

**DOI:** 10.3390/antiox10071083

**Published:** 2021-07-05

**Authors:** Anallely López-Yerena, Anna Vallverdú-Queralt, Rosa M. Lamuela-Raventós, Elvira Escribano-Ferrer

**Affiliations:** 1 Department of Nutrition, Food Science and Gastronomy XIA Faculty of Pharmacy and Food Sciences, Institute of Nutrition and Food Safety (INSA-UB), University of Barcelona, 08028 Barcelona, Spain; naye.yerena@gmail.com (A.L.-Y.); avallverdu@ub.edu (A.V.-Q.); lamuela@ub.edu (R.M.L.-R.); 2 CIBER Physiopathology of Obesity and Nutrition (CIBEROBN), Institute de Salud Carlos III, 28029 Madrid, Spain; 3 Biopharmaceutics and Pharmacokinetics Unit, Department of Pharmacy and Pharmaceutical Technology and Physical Chemistry, Faculty of Pharmacy and Food Sciences, Institute of Nanoscience and Nanotechnology (IN2UB), University of Barcelona, 08028 Barcelona, Spain; 4 Pharmaceutical Nanotechnology Group I+D+I Associated Unit to CSIC, University of Barcelona, 08028 Barcelona, Spain

**Keywords:** biotransformation, extra virgin olive oil, hydroxytyrosol, phenolic compound, secoiridoid

## Abstract

The purpose of this work was to study the distribution of oleacein (OLEA) and its metabolites in rat plasma and different tissues, namely brain, heart, kidney, liver, lung, small intestine, spleen, stomach, skin, and thyroid, following the acute intake of a refined olive oil containing 0.3 mg/mL of OLEA. For this purpose, a distribution kinetics study was carried out. The plasma and tissues were collected at 1, 2, and 4.5 h after the intervention, and analyzed by LC-ESI-LTQ-Orbitrap-MS. Unmetabolized OLEA was detected in the stomach, small intestine, liver, plasma and, most notably, the heart. This finding may be useful for the development of new applications of OLEA for cardiovascular disease prevention. Noteworthy are also the high levels of hydroxytyrosol (OH-TY) and OLEA + CH_3_ found in the small intestine, liver, and plasma, and the detection of nine OLEA metabolites, five of them arising from conjugation reactions. Liver, heart, spleen, and lungs were the target tissues where the metabolites were most distributed. However, it is important to note that OH-TY, in our experimental conditions, was not detected in any target tissue (heart, spleen, thyroids, lungs, brain, and skin). These results shed further light on the metabolism and tissue distribution of OLEA and contribute to understanding the mechanisms underlying its effect in human health.

## 1. Introduction

Oleacein (OLEA), also termed 3,4-DHPEA-EDA, plays a significant role in the organoleptic qualities of extra virgin olive oil (EVOO), and its antioxidant properties improve the oil’s shelf life [1,2]. It is one of the most abundant compounds in EVOO and the concentrations of this secoiridoid can reach up to 1834 ± 110 mg/kg [3,4]. OLEA is also reported to have biological and pharmacological activities with wide-ranging health benefits. In 2012, an in vitro study showed that OLEA may offer far more protection against reactive oxygen/nitrogen species-induced injury of human biomolecules than its parent compound oleuropein [5]. It offers protection against cardiovascular diseases, decreasing the risk of atherosclerosis [6,7,8], ischemic stroke [9], and hypertension [10]. Anti-cancer effects have also been reported, including antiproliferative and apoptosis-inducing activity in leukemia cells [11] and reduction of viability and migration of non-melanoma skin cancer cells [12]. An in vitro study found that OLEA has significant anti-tumor activity by promoting cell cycle arrest and apoptosis [13]. Moreover, OLEA has been associated with healthy aging in both in vitro and in vivo studies [14]. Anti-inflammatory properties provide OLEA with a protective effect against several metabolic abnormalities, including dyslipidemia, insulin resistance, and glucose intolerance [15,16].

Despite numerous studies on the biological activity of OLEA, its tissue distribution is not well documented. To date, only one study has focused on elucidating the absorption of OLEA, through simultaneous sampling from the luminal perfusate and mesenteric blood, and its metabolism during enterocytic transfer in the ileum [17]. In addition, the stability of OLEA under low pH conditions and permeability in Caco-2 cells, and in isolated rat intestine has been studied [18] as well as its fecal microbial metabolism in vivo [19]. The distribution and accumulation of EVOO polyphenols [20] and oleocanthal [21] has also been determined in rat tissues. The health-enhancing properties of OLEA cannot be fully understood without further analysis of its absorption, tissue distribution, metabolism, and excretion.

In this context, we carried out a qualitative and quantitative evaluation of OLEA and its metabolites in a range of biological samples taken from rats at different times (0, 1, 2, and 4.5 h) following the administration of refined olive oil (ROO) containing OLEA. For the first time, OLEA and its metabolites were studied in organs of the digestive system (stomach, small intestine, and liver), excretory tissue (kidney), and several target tissues (heart, spleen, thyroids, lungs, brain, and skin).

## 2. Materials and Methods

### 2.1. Chemicals and Reagents

The reference standard OLEA (≥90%) was purchased from Toronto Research Chemicals (North York, ON, Canada) and hydroxytyrosol (OH-TY) was obtained from Extrasynthese (Genay, France). The reagents methanol, acetonitrile (ACN), and formic acid were acquired from AppliChem, Panreac Quimica SLU (Barcelona, Spain). Ultrapure water was obtained using a Milli-Q purification system (Millipore, Bedford, MA, USA).

### 2.2. Animals

In this study, male Sprague–Dawley rats (body weight 227 ± 11 g) were obtained from Envigo RMS Spain SL (Sant Feliu de Codines, Barcelona, Spain). The animals were acclimated for at least 1 week before the initiation of dosing with free access to food and water. They were kept at 22 ± 2 °C in an air-conditioned room on a 12-h light–12-h dark cycle. Before the experiments, the animals were fasted overnight with water available ad libitum. The experiments were performed in compliance with the Animal Experimentation Ethics Committee of the University of Barcelona, Spain and were approved by the Generalitat de Catalunya (trial no. CEEA 124/16 and no. 6435, respectively). 

### 2.3. Refined Olive Oil

Before the experiment, the ROO (used as a vehicle to dissolve OLEA for intragastric administration) was analyzed by LC-ESI-LTQ-Orbitrap-MS to make sure it was free of phenolic compounds. The liquid–liquid extraction of phenolic compounds in the ROO was performed following previously described procedures [22]. The chromatographic and mass spectrometer conditions were the same as used for the identification of metabolites (Section 2.6). Once the absence of phenolic compounds was confirmed, the OLEA was added to obtain a final concentration of 0.3 mg/mL. This concentration was chosen based on its concentration in commercial EVOO (around 300 mg/kg), and the daily ingestion recommended by the European Food Safety Authority of at least 5 mg of hydroxytyrosol and its derivatives per 20 g of olive oil [23]. 

### 2.4. Dosage Information

The rats were divided into three different groups according to the time of euthanasia: 1 h (*n* = 4), 2 h (*n* = 5), and 4.5 h (*n* = 4). At 0 h, ROO loaded with OLEA standard was supplied to the rats (2.5 mL/300 g body weight) by intragastric administration. Additionally, a control group of rats was maintained under fasting conditions without olive oil ingestion and then similarly euthanized. The gavage volume was established following a good practice guide for substance administration [24] and was lower than the maximum volume of lipid vehicles per kg of rat body weight that prevents elevated plasma corticosterone levels [25].

The rats were anesthetized with isoflurane (IsoFlo, veterinaria Esteve, Barcelona, Spain) and euthanized by exsanguinations at the aforementioned time points after the ROO ingestion. Blood samples were collected in EDTA tubes, centrifuged to obtain the plasma (3000× *g*, 10 min at 4 °C), and stored at −80 °C until analysis. The gastrointestinal tract (including stomach and small intestine), heart, spleen, thyroids, lungs, brain, kidney, and skin were removed from rats at the designated time points. All tissues were washed with saline solution (NaCl 0.9%) and dried with filter paper. In the case of the intestine, the portion removed was 15 cm from 2 cm posterior to the pyloric sphincter, and the intestine was also washed internally by perfusing the isotonic saline solution several times. Rat tissues were accurately weighed and stored at −80 °C until analysis. 

### 2.5. Sample Pre-Treatment for OLEA Metabolite Analysis

For extraction, all samples were slowly thawed in ice. To avoid compound oxidation, the samples were handled in a room with filtered light and kept on ice throughout. The clean-up of the biological matrix (protein precipitation) was performed using a previously developed method [21].

#### 2.5.1. Plasma

Plasma samples were centrifuged for 10 min (11,000× *g*, 4 °C). Briefly, 100 µL of each sample was deproteinized with ice-cold ACN acidified with formic acid (2%) in a 1:5 (*v*/*v*) ratio. The samples were then homogenized for 1 min and kept at −20 °C in a freezer for 20 min. After that, samples were centrifuged (11,000× *g*, 4 °C, 10 min) and finally, 100 µL of each organic phase was transferred to vials for analysis.

#### 2.5.2. Tissues

A small tissue disruptor (T 10 basic ULTRA-TURRAX^®^, IKA laboratory technology, Staufen, Germany) was used to homogenize the thawed tissues in a 1:1 ratio (*w*/*v*) with water:ACN (1:1 (*v*/*v*) with 0.1% ascorbic acid). The samples were then sonicated for 5 min in an ice bath, shaken for 1 min (Vortex-Genie 2) and centrifuged for 10 min (11,000× *g*, 4 °C). To precipitate the proteins, a volume of 100 µL of the upper layer was blended with cold ACN acidified with formic acid (2%) in a 1:3 (*v*/*v*) ratio. After that, the samples were homogenized for 1 min and kept at −20 °C in a freezer for 20 min. Finally, the samples were centrifuged for 10 min (11,000× *g*, 4 °C) and 100 µL of supernatant was transferred to vials for analysis.

### 2.6. Instruments and Analytical Conditions

The metabolic profile was determined by analyzing the samples with LC-ESI-LTQ-Orbitrap-MS. The equipment was an Accela chromatograph (Thermo Scientific, Hemel Hempstead, UK) with a quaternary pump, a photodiode array detector (PDA) and a thermostated autosampler coupled with a LTQ Orbitrap Velos mass spectrometer (Thermo Scientific, Hemel Hempstead, UK) equipped with an ESI source in negative mode.

Chromatographic separation was performed using an AcquityTM UPLC^®^ BEH C_18_ Pre-Column (2.1 × 5 mm, i.d., 1.7 µm particle size) and an AcquityTM UPLC^®^ BEH C_18_ Column (2.1 × 100 mm, i.d., 1.7 µm particle size) (Waters Corporation^®^, Ireland) maintained at 50 °C. The mobile phases consisted of methanol (A) and H_2_O (B), both with 0.1% of formic acid. The elution was performed by means of a decreasing linear gradient (*v*/*v*) of B (t (min), %B), as follows: (0, 100); (2, 100); (6, 46.4); (8, 0); (9, 0); (9.1, 100); (11, 100). The injection volume and the flow rate were set to 5 µL and 0.6 mL/min, respectively. 

The instrumental conditions for the LTQ Orbitrap Velos mass spectrometer were the same as reported in our previous study [17]. The identification of OLEA and OH-TY was achieved using their respective commercially available standards. The identification of the other metabolites was based on chromatographic elution time, chemical composition, and MS/MS fragmentation pattern, also as performed previously [17].

For quantification purposes, the calibration curves were prepared with OLEA in plasma and each blank isolated tissue (brain, heart, kidney, liver, lung, small intestine, spleen, stomach, skin, and thyroid) in concentrations of 0.1 to 3 µg/mL. All calibration curves presented R^2^ > 0.97. The results of all OLEA metabolites were calculated using the peak area of the parent molecule and were expressed as nmol OLEA equivalents/mL or nmol OLEA equivalents/g tissue.

### 2.7. Data Analysis

All statistical analyses were performed with Statgraphics Centurion XVI software (Statpoint Technologies Inc., Warrenton, VA, USA). Data are presented as mean and standard deviation (SD). The assumption of normalization was checked with standardized bias and standardized kurtosis. Statistical differences in the concentration of metabolites and OLEA at different post-administration times (1, 2, and 4.5 h) for each compound and tissue were evaluated using a one-way ANOVA, followed by the LSD post-hoc test. Differences were considered statistically significant at *p* < 0.05.

## 3. Results and Discussion

### 3.1. Pre-Systemic Metabolism

The concentrations of OLEA and its derivatives in the stomach, small intestine, and liver expressed as nmol OLEA equivalents/g tissue are presented in Table 1. The highest concentration (C_max_) of OLEA in the stomach (8.73 ± 0.29 OLEA equivalents/g tissue) was found at 1 h after acute ingestion of ROO, and it had significantly decreased by 16% at 2 h and by 34% at 4.5 h (*p* < 0.05). One explanation for this reduction is gastric emptying, as OLEA was observed in the small intestine at 1 h post-ingestion. Another possible cause is the acidic environment of the stomach and/or the presence of nonlipolytic carboxylester hydrolases, which can contribute to a hydrolysis of OLEA, resulting in OH-TY and elenolic acid (EA) [26]. Contradictory results about the gastric stability of OLEA have been reported. In a recent in vivo study with rats by Pinto et al. [18], OLEA was found to undergo a complete degradation in the gastric region, and only a partial hydrolysis after incubation at pH 2.0 (releasing OH-TY), with about 67% of OLEA remaining after 4 h. A partial hydrolysis was also observed in our study, as the amount of OLEA reaching the small intestine was lower than in the stomach at the same time point (Table 1). The C_max_ of OLEA in the small intestine was reached after 1 h of ROO intake, decreasing successively thereafter (by 25% and 77% at 2 h and 4.5 h, respectively) (*p* < 0.05). This reduction is mainly attributable to its good permeability through the intestinal epithelium, as reported recently [17], and transport to the liver (where notably OLEA was detected only at 1 h post-ingestion). The low concentration of OLEA in the liver vs. the small intestine is explained by the partial intestinal metabolism of OLEA during the absorption [17]. 

The metabolites OH-TY and EA released in the stomach were transported to the small intestine, where they were absorbed. As shown in Table 1, similar amounts of these compounds were found in the liver. Both compounds are weak basic organic aromatic molecules with very low molecular weights (<250 g/mol) and moderate lipophilicity (logP ~ 0.9), which favors their absorption through passive mechanisms [26,27]; their detection in plasma confirmed their absorption (Table 2). Current data suggest OH-TY is the only biologically active derivative of OLEA [28,29], so the detection of free OH-TY in the liver is noteworthy, pointing to its potential application in the prevention of certain hepatic diseases. Our results are in line with other studies that demonstrate that OH-TY can reach the liver [30]. Our results, with regard to the OH-TY concentration in stomach at 1 h, were similar to those reported in another study (8.75 ± 1.95 nmols/g digesta) [19], demonstrating the degradation of OLEA at the stomach level.

As shown in Table 1, the metabolite arising from the hydrogenation of OLEA (OLEA + H_2_) was detected in the stomach and small intestine, with the C_max_ in both tissues at 2 h (*p* < 0.05). In previous studies, this metabolite was not identified in the stomach, small intestine or liver [19,20], although it has been detected in human urine [31] and rat plasma [17]. The hydrogenation of an aldehyde could be attributed to aldo–keto reductases (AKRs) that primarily reduce aldehydes and ketones to primary and secondary alcohols, respectively [32]. In our study, the absence of the hydrogenated metabolite in the liver could be attributed to other biotransformations once it reaches this tissue, such as glucuronidation. It is well known that conjugation reactions lead to the formation of a covalent bond joining a functional group in the parent compound or phase I metabolite [33].

The metabolite OLEA + H_2_O was detected in the stomach, small intestine, and liver samples. The C_max_ in the two first tissues was reached at 1 h, and in the liver at 2 h (*p* < 0.05), reflecting the time required for absorption and transport. These results support those described by García-Villalba et al. [31] and López-Yerena et al. [17], who identified this metabolite in human urine and rat plasma, respectively. However, in other studies of secoiridoid metabolism, this metabolite has not been quantified [18,19,20,34].

Regarding OLEA derivatives arising from conjugation reactions, a methylated metabolite (OLEA + CH_3_) was detected in the stomach, small intestine, and the liver, with higher concentrations in the latter two organs. In the liver, it was the metabolite with the highest concentration. The catechol-*O*-methyltransferase (COMT) enzymes, most active in the liver and kidneys, but also in the pylorus of the stomach and in the small intestine [35], are responsible for the methylation of available OLEA. Glycoconjugated metabolites, on the other hand, require the activity of uridine-disphosphate glucuronosyl transferase (UGT). Although these enzymes are present in the stomach [36], and UGT1A8 and UGT1A10 are exclusively expressed in the small intestine [37], the liver is the main site of glucuronidation [36], which could explain why we found metabolites with a glucuronide acid only in the liver and not in the stomach and small intestine. Regarding metabolites not derived from hydrolysis reactions, their concentrations were very low (or even absent) in the stomach and increased in the intestine and liver, in accordance with the higher qualitative and quantitative enzyme endowment of these organs. These metabolites were also detected in the intestinal lumen and ileal tissue by López-Yerena [17]. The fact that we did not find the metabolites OLEA+ H_2_O + CH_3_, OLEA + H_2_ + glucu and OLEA + H_2_O + CH_3_ + glucu in the small intestine (Table 1), yet they were detected in the aforementioned study (at very low concentrations), can be mainly attributed to methodological differences (in situ vs. oral administration). 

### 3.2. Plasma

Table 2 shows the concentration of OLEA and its phase I and II metabolites detected in rat plasma after the intragastric administration of OLEA in ROO. As can be observed, OLEA reached the systemic circulation unchanged, being found in the plasma at all three sampling times. The highest concentration was at 1 h post-ROO intake (1.37 ± 0.20 nmol OLEA equivalents/mL), and some remained at 4.5 h (0.67 ± 0.10 nmol OLEA equivalents/mL). Our results are in accordance with those of Silva et al. [34] who identified unmetabolized OLEA in human plasma after EVOO intake. Recently, OLEA was also detected in mesenteric rat plasma after in situ perfusion of the small intestine [17]; this method avoided the pass through the stomach where hydrolysis can potentially take place, but the OLEA would still have to pass through the liver to reach the systemic circulation. In contrast with the present results, OLEA was not identified in rat plasma after the administration of a phenolic extract from olive cake [20] or a diet supplemented with OLEA [19]. The presence of OLEA in plasma at all sample times, unlike in the liver, where it was only detected at 1 h, could be due to an in situ deconjugation in red blood cells, as suggested for sulphates and glucuronidates of OH-TY [38].

The metabolites circulating in plasma at the highest concentrations were methylated OLEA (OLEA+ CH_3_) and OH-TY. The C_max_ of methylated OLEA was highest at 2 h post-ingestion of ROO (3.50 ± 0.18 nmol OLEA equivalents/mL), representing 25% of the total metabolites identified at this time point (*p* < 0.05). The second most abundant, OH-TY, represented 20, 22% and 17% of the total metabolites at 1, 2, and 4.5 h, respectively. Our results are similar to those of Serra et al. [20], who linked the high percentage of total phenyl alcohol metabolites (e.g., OH-TY and tyrosol) in plasma with the high content of OLEA in the administered olive cake extract, which acted as a source of OH-TY. Contrary results were observed by López de las Hazas et al. [39], who did not detect free OH-TY in human plasma after the acute intake of an olive oil phenolic extract. Differences in metabolism between rodents and humans may be the reason for these differences in results [40]. Other reasons may include: (i) the vehicle, (ii) individual genomic profile (affecting enzymatic activity involved in the digestion and metabolism processes), and (iv) polymorphism of conjugation enzymes or individual variations in digestive enzymes or bile salts [34,40,41].

The detection of free OH-TY in plasma may shed light on the health benefits of the phenolic content of EVOO. Interest in OH-TY has grown since 2012, when the European Food Safety Agency (EFSA) Panel on Dietetic Products, Nutrition, and Allergies (NDA) established a cause-and-effect relationship between the consumption of olive oil polyphenols and protection of LDL particles from oxidative damage [28]. Based on this scientific opinion, “Olive oil polyphenols contribute to the protection of blood lipids from oxidative stress” was included in the list of authorized health claims in the European Union [29], being the only relating polyphenols with health. 

Other metabolites arising from phase I (EA, OLEA + H_2_ and OLEA + H_2_O) and phase II reactions (OLEA + H_2_O + CH_3_, OLEA + H_2_ + glucu, OLEA + H_2_O + glucu, OLEA + H_2_O + CH_3_ + glucu) were also detected in plasma samples. For all metabolites, the C_max_ was reached at 2 h after ROO consumption. In another study, OLEA + H_2_O + CH_3_ + glucu was also identified in human plasma [34].

### 3.3. Accumulation and Distribution of OLEA Derivatives in Rat Tissues

To date, only two studies have focused on the metabolism and distribution of secoiridoids, including OLEA, in target tissues of rats [19,20]. The vehicles they used to administer the phenolic compounds (pellets containing a secoiridoid extract and a phenolic extract from olive cake dispersed in water, respectively) could be seen as a drawback when attempting to elucidate the health effects of EVOO. In our study, ROO was chosen as the vehicle to better simulate the conditions of EVOO consumption. In addition, the metabolite profile was determined by UPLC coupled with high-resolution mass spectrometry (HRMS), using Orbitrap technology to overcome the limitations of the low-resolution MS techniques (triple quadrupole) of the two previous studies [19,20,42]. The main results are shown in Table 3. It is important to note that both OH-TY and EA as well as their metabolites were not detected in heart, spleen, thyroids, lungs, brain, kidney, and skin.

#### 3.3.1. Heart

The heart was the only organ where unmetabolized OLEA was detected without significant differences in concentration between the time points (*p* > 0.05) (0.44 ± 0.07, 0.45 ± 0.04 and 0.44 ± 0.06 nmol OLEA equivalents/g tissue, at 1, 2, and 4.5 h, respectively). The effect of OLEA on the progression of different types of cardiovascular diseases has been studied in vitro. These include biological effects, such as the ability to regulate low-density lipoprotein (LDL) oxidation and myeloperoxidase (MPO) activity, to reduce the expression of adhesion molecules through angiotensin II production, and to confer protection to erythrocytes from oxidative hemolysis [5,6,7,8,9]. Although the concentrations detected in the heart (≤0.5 nmol OLEA equivalents/g tissue) were very low compared with the results of in vitro studies (50 and 100 mM [6] and 10–20 mM [7]), this is the first demonstration that OLEA is able to reach the heart, where it may exert a beneficial effect. However, more studies are necessary to determine if the concentration detected in our study would be sufficient for OLEA to play a preventive and curative role against cardiovascular injury. In addition to OLEA, seven metabolites were also detected (Table 3). 

#### 3.3.2. Spleen, Lungs and Thyroids

Seven metabolites in the spleen and lungs and six in thyroids were detected (Table 3), all with a C_max_ at 2 h after ROO ingestion. The activity of metabolizing enzymes in the endothelial cells of lungs [43,44] and their expression in macrophages and lymphocytes in the white pulp region of the spleen [20] could explain the presence of these metabolites in these tissues. 

#### 3.3.3. Brain

The main metabolites found in the brain were from phase II reactions. The only phase I metabolite quantified was the hydrated form of OLEA vs. five conjugated metabolites detected in greater number and concentration. After 1 h of ROO ingestion, the main metabolites identified were OLEA + CH_3_, OLEA + H_2_ + glucu, and OLEA + H_2_O + glucu. Neurodegenerative diseases normally begin in the elderly, caused by a significant accumulation of reactive oxygen species in the brain due to unknown causes. In neurodegeneration, mitochondrial dysfunction, neuroinflammation, and in some pathologies, protein aggregation and neuronal death culminate in brain dysfunction [45]. Recently, Gutiérrez-Miranda et al. [46] suggested that OLEA may provide protective effects against autoimmune encephalomyelitis by reducing leukocyte infiltration into the central nervous system, preventing the formation of inflammatory mediators, and inhibiting the elevation of the oxidative stress status. According to the results of our study, in which free OLEA was not detected in the brain, more research is needed to determine the bioactivity of the main metabolites at the physiological concentrations achieved after regular consumption of EVOO, or the potential intracellular deconjugation of the metabolites in the target tissue. 

Additionally, it is important to note that although OH-TY has been shown to be capable of reaching the brain [45], it was not detected in our study. Differences in administered doses may explain these differences (5 mg/kg/day during 21 days vs. 0.3 mg/mL in an acute study).

#### 3.3.4. Kidneys

As shown in Table 3, only conjugated metabolites were detected in the kidneys, which is the only organ where the highest concentration of metabolites was found at 4.5 h post-ROO consumption (*p* < 0.05). As kidneys are an elimination organ, hydrophilic metabolites are likely to accumulate there for excretion. At 4.5 h, the main metabolite identified was OLEA + H_2_O + glucu, with a C_max_ of 0.91 ± 0.05 nmol OLEA equivalents/g tissue. The wide range of metabolites detected indicates that renal excretion is a major pathway of OLEA metabolite elimination in rats. The presence of metabolites in the kidneys could also be attributed to the activity of metabolic enzymes (CYP and UGT). By regulating renal drug and chemical metabolic clearance, they modulate intra-renal exposure to compounds as well as the activity of physiological mediators [47]. In 2010, Garcia Villalba et al. [31] identified the same metabolites in human urine 2 h after the intake of olive oil.

#### 3.3.5. Skin

The skin is the least studied tissue in distribution studies after a phenolic compound ingestion. In a recent study of oleocanthal distribution, four metabolites were detected in the skin [21], whereas we identified only OLEA + CH_3_. The concentration of this metabolite increased over time: 0.09 ± 0.01, 0.15 ± 0.02 and 0.25 ± 0.02 nmol OLEA equivalents/g tissue at 1, 2, and 4.5 h, respectively, achieving a C_max_ at 4.5 h post-ingestion (results not included in the Table 3). The skin is a relatively poorly perfused tissue compared to well-perfused tissues like the lungs, heart, and thyroid gland, and therefore the compounds reach it more slowly through the blood circulation [48]. Despite the detection of only one metabolite in the skin, this finding may help to shed new light on the mechanisms underlying EVOO phenolic compound activity in the body, specifically in the area of skin disorder prevention.

## 4. Conclusions

In this work, the distribution of OLEA and derived metabolites in rat plasma and ten organs/tissues was investigated using an LC-ESI-LTQ-Orbitrap-MS system. In our study, a comprehensive study of plasma, stomach, small intestine, liver, heart, spleen, thyroids, lungs, brain, kidney, and skin was performed after a single oral administration of OLEA in ROO (by gastric gavage).

OLEA was detected in its free form in the stomach, and was also found to be hydrolyzed resulting in OH-TY and EA. Unmetabolized OLEA was also detected in the small intestine, liver, plasma, and most interestingly, the heart. This finding may be useful for the development of new applications of OLEA to prevent cardiovascular diseases. The results may also shed light on why OLEA is active in vivo, even if its blood concentration is low. Furthermore, the high levels of OH-TY and OLEA + CH_3_ found in the small intestine, liver, and plasma are noteworthy. Regarding the metabolite profiles in tissues, up to seven metabolites were identified. The liver, heart, spleen, and lungs were the target tissues where the metabolites were detected to be greater. It is important to note that OH-TY did not reach any of these tissues. The presence of parent and/or OLEA metabolites in rat tissues could explain the myriad of health benefits attributed to EVOO consumption. In summary, after the oral administration of OLEA, its metabolites were more widely distributed in tissues and plasma than the parent compound.

## Figures and Tables

**Table 1:** Concentration of OLEA and phase I and phase II metabolites in the stomach, small intestine, and liver, expressed as nmol OLEA equivalents/g tissue. Results are shown as the mean ± standard deviation.

Metabolite (nmol OLEA Equivalents/g Tissue)	Time (h)	Stomach	Small Intestine	Liver
OLEA	1	8.73 ± 0.29 ^a^	2.24 ± 0.14 ^a^	0.28 ± 0.02
	2	7.39 ± 0.25 ^b^	1.69 ± 0.25 ^b^	n.d.
	4.5	5.77 ± 0.54 ^c^	0.51 ± 0.10 ^c^	n.d.
OH-TY	1	13.37 ± 1.27 ^a^	4.71 ± 0.32 ^a^	3.34 ± 0.08 ^b^
	2	7.81 ± 0.69 ^b^	3.79 ± 0.29 ^b^	3.83 ± 0.21 ^a^
	4.5	5.23 ± 0.37 ^c^	2.70 ± 0.21 ^c^	2.51 ± 0.07 ^c^
EA	1	7.53 ± 0.56 ^a^	1.81 ± 0.05 ^a^	1.07 ± 0.06 ^b^
	2	4.07 ± 0.36 ^b^	0.67 ± 0.08 ^b^	1.34 ± 0.08 ^a^
	4.5	1.81 ± 0.13 ^c^	0.16 ± 0.02 ^c^	0.76 ± 0.02 ^c^
OLEA + H_2_	1	1.34 ± 0.01 ^a^	5.31 ± 0.48 ^a^	n.d.
	2	0.82 ± 0.01 ^b^	3.01 ± 0.35 ^b^	n.d.
	4.5	0.81 ± 0.01 ^b^	2.16 ± 0.10 ^c^	n.d.
OLEA + H_2_O	1	1.90 ± 0.18 ^a^	1.29 ± 0.14 ^a^	0.33 ± 0.02 ^b^
	2	1.66 ± 0.12 ^a^	0.96 ± 0.06 ^b^	0.77 ± 0.01 ^a^
	4.5	0.80 ± 0.03 ^b^	0.53 ± 0.01 ^c^	0.26 ± 0.04 ^c^
OLEA + CH_3_	1	0.46 ± 0.05 ^a^	3.69 ± 0.19 ^a^	3.88 ± 0.45 ^b^
	2	0.41 ± 0.01 ^a^	2.99 ± 0.46 ^b^	5.79 ± 0.36 ^a^
	4.5	0.26 ± 0.00 ^b^	2.26 ± 0.22 ^c^	1.90 ± 0.24 ^c^
OLEA + H_2_O + CH_3_	1	n.d.	n.d.	1.37 ± 0.12 ^b^
	2	n.d.	n.d.	1.78 ± 0.07 ^a^
	4.5	n.d.	n.d.	1.10 ± 0.07 ^c^
OLEA + H_2_ + glucu	1	n.d.	n.d.	0.77 ± 0.05 ^b^
	2	n.d.	n.d.	1.18 ± 0.06 ^a^
	4.5	n.d.	n.d.	0.46 ± 0.05 ^c^
OLEA+ H_2_O + CH_3_ + glucu	1	n.d.	n.d.	0.38 ± 0.03 ^b^
	2	n.d.	n.d.	0.69 ± 0.04 ^a^
	4.5	n.d.	n.d.	0.26 ± 0.01 ^c^

Different letters in each metabolite and tissue indicate significant differences in concentration between 1, 2, and 4.5 h (*p* < 0.05). n.d. not detected.

**Table 2:** Mean concentration of OLEA and its phase I and phase II metabolites ± standard deviation in plasma. Results are expressed as nmol OLEA equivalents/mL of plasma.

Metabolites (nmol OLEA Equivalents/mL)	Time (h)		
	1	2	4.5
OLEA	1.37 ± 0.20 ^a^	0.65 ± 0.07 ^b^	0.67 ± 0.10 ^b^
OH-TY	2.11 ± 0.10 ^b^	3.02 ± 0.16 ^a^	1.62 ± 0.14 ^c^
EA	0.43 ± 0.03 ^b^	0.76 ± 0.04 ^a^	0.16 ± 0.02 ^c^
OLEA + H_2_	0.87 ± 0.02 ^b^	1.08 ± 0.02 ^a^	0.87 ± 0.02 ^b^
OLEA + H_2_O	0.91 ± 0.03 ^b^	1.14 ± 0.02 ^a^	0.84 ± 0.02 ^c^
OLEA + CH_3_	2.52 ± 0.24 ^b^	3.50 ± 0.18 ^a^	2.80 ± 0.22 ^b^
OLEA + H_2_O + CH_3_	1.04 ± 0.01^b^	1.35 ± 0.05 ^a^	0.91 ± 0.02 ^c^
OLEA + H_2_ + glucu	0.88 ± 0.04 ^b^	1.10 ± 0.04 ^a^	0.71 ± 0.02 ^c^
OLEA + H_2_O + glucu	1.26 ± 0.03 ^b^	1.44 ± 0.04 ^a^	1.03 ± 0.03 ^c^
OLEA + H_2_O + CH_3_ + glucu	0.57 ± 0.01 ^b^	0.65 ± 0.01 ^a^	0.50 ± 0.01 ^b^

Different letters in each metabolite and tissue indicate significant differences in concentration between 1, 2 and 4.5 h (*p* < 0.05).

**Table 3:** Mean ± standard deviation of tissue concentration of metabolites, expressed as nmol OLEA equivalents/g tissue, following the acute intake of OLEA (0.3 mg/mL) in ROO.

Metabolites (nmol OLEA Equivalents/g Tissue)	Time (h)	Heart	Spleen	Thyroids	Lungs	Brain	Kidney
OLEA	1	0.44 ± 0.07 ^a^	n.d.	n.d.	n.d.	n.d.	n.d.
	2	0.45 ± 0.04 ^a^	n.d.	n.d.	n.d.	n.d.	n.d.
	4.5	0.44 ± 0.06 ^a^	n.d.	n.d.	n.d.	n.d.	n.d.
OLEA + H_2_	1	0.17 ± 0.02 ^b^	0.30 ± 0.05 ^c^	0.10 ± 0.01 ^c^	0.49 ± 0.04 ^c^	n.d.	n.d.
	2	0.30 ± 0.04 ^a^	0.54 ± 0.03 ^a^	0.24 ± 0.01 ^a^	0.70 ± 0.05 ^a^	n.d.	n.d.
	4.5	0.18 ± 0.01 ^b^	0.34 ± 0.02 ^b^	0.12 ± 0.01 ^b^	0.52 ± 0.01 ^b^	n.d.	n.d.
OLEA + H_2_O	1	0.07 ± 0.01 ^b^	0.42 ± 0.03 ^b^	0.12 ± 0.02 ^b^	0.49 ± 0.02 ^b^	0.13 ± 0.03 ^b^	n.d.
	2	0.17 ± 0.02 ^a^	0.61 ± 0.07 ^a^	0.24 ± 0.02 ^a^	1.02 ± 0.12 ^a^	0.22 ± 0.02 ^a^	n.d.
	4.5	0.07 ± 0.01 ^b^	0.42 ± 0.02 ^b^	0.13 ± 0.03 ^b^	0.42 ± 0.05 ^b^	0.10 ± 0.01 ^b^	n.d.
OLEA + CH_3_	1	0.20 ± 0.02 ^b^	0.31 ± 0.03 ^b^	0.21 ± 0.02 ^b^	0.35 ± 0.03 ^b^	0.32 ± 0.03 ^b^	0.17 ± 0.01 ^b^
	2	0.36 ± 0.03 ^a^	0.39 ± 0.04 ^a^	0.31 ± 0.02 ^a^	0.45 ± 0.04 ^a^	0.52 ± 0.04 ^a^	0.20 ± 0.02 ^b^
	4.5	0.25 ± 0.03 ^b^	0.27 ± 0.03 ^b^	0.24 ± 0.01 ^b^	0.31 ± 0.02 ^b^	0.30 ± 0.03 ^b^	0.34 ± 0.04 ^a^
OLEA + H_2_O + CH_3_	1	0.11 ± 0.01 ^b^	0.18 ± 0.03 ^c^	0.14 ± 0.01 ^b^	0.62 ± 0.06 ^b^	0.17 ± 0.02 ^b^	0.13 ± 0.02 ^c^
	2	0.18 ± 0.03 ^a^	0.68 ± 0.03 ^a^	0.22 ± 0.03 ^a^	0.84 ± 0.04 ^a^	0.35 ± 0.02 ^a^	0.23 ± 0.03 ^b^
	4.5	0.08 ± 0.01 ^c^	0.44 ± 0.04 ^b^	0.09 ± 0.09 ^c^	0.48 ± 0.04 ^c^	0.15 ± 0.02 ^b^	0.43 ± 0.06 ^a^
OLEA + H_2_ + glucu	1	0.13 ± 0.02 ^b^	0.08 ± 0.01 ^b^	0.17 ± 0.02 ^b^	0.24 ± 0.01 ^b^	0.28 ± 0.03 ^b^	0.10 ± 0.02 ^c^
	2	0.20 ± 0.01 ^a^	0.18 ± 0.01 ^a^	0.28 ± 0.01 ^a^	0.37 ± 0.01 ^a^	0.40 ± 0.03 ^a^	0.35 ± 0.13 ^b^
	4.5	0.11 ± 0.01 ^b^	0.08 ± 0.01 ^b^	0.07 ± 0.01 ^c^	0.11 ± 0.02 ^c^	0.09 ± 0.02 ^c^	0.64 ± 0.03 ^a^
OLEA + H_2_O + glucu	1	0.42 ± 0.07 ^a^	0.30 ± 0.03 ^b^	0.21 ± 0.02 ^b^	0.32 ± 0.02 ^b^	0.28 ± 0.03 ^b^	0.20 ± 0.03 ^b^
	2	0.43 ± 0.06 ^a^	0.43 ± 0.03 ^a^	0.35 ± 0.05 ^a^	0.45 ± 0.03 ^a^	0.41 ± 0.03 ^a^	0.25 ± 0.04 ^b^
	4.5	0.17 ± 0.02 ^b^	0.13 ± 0.02 ^c^	0.01 ± 0.02 ^c^	0.15 ± 0.02 ^c^	0.10 ± 0.02 ^c^	0.91 ± 0.05 ^a^
OLEA + H_2_O + CH_3_ + glucu	1	0.05 ± 0.00 ^c^	0.11 ± 0.02 ^b^	n.d.	0.06 ± 0.01 ^b^	0.04 ± 0.00 ^b^	0.05 ± 0.00 ^c^
	2	0.20 ± 0.01 ^a^	0.42 ± 0.04 ^a^	n.d.	0.10 ± 0.02 ^a^	0.08 ± 0.01 ^a^	0.14 ± 0.02 ^b^
	4.5	0.13 ± 0.01 ^b^	0.05 ± 0.00 ^c^	n.d.	0.04 ± 0.01 ^b^	0.05 ± 0.01 ^b^	0.40 ± 0.02 ^a^

Different letters in each metabolite and tissue indicate significant differences in concentration between 1, 2, and 4.5 h (*p* < 0.05). n.d. not detected.

## Data Availability

Data is contained within the article. The data presented in this study are available in Table 1, Table 2 and Table 3.
